# Editorial: Advances in basic and applied research in photoplethysmography

**DOI:** 10.3389/fphys.2024.1415049

**Published:** 2024-05-16

**Authors:** John Allen, Panicos A. Kyriacou

**Affiliations:** ^1^ Research Centre for Intelligent Healthcare, Coventry University, Coventry, United Kingdom; ^2^ Research Centre for Biomedical Engineering, University of London, London, United Kingdom

**Keywords:** photoplethysmography, pulse wave analysis, diagnostic, physiology, optical, machine learning, cardiovascular

## Introducing photoplethysmography

Welcome to this Research Topic in Frontiers in Physiology, focusing on Advances in Basic and Applied Research in Photoplethysmography.

Photoplethysmography (PPG) is a low-cost and simple vascular optics technique that can be used to detect blood volume changes in the microvascular bed of tissue with each heartbeat (Allen, 2007; Kyriacou and Allen, 2021). The popularity of this Research Topic area can be attributed to the realization that PPG has important implications for a wide range of applications including assessment of the cardiovascular system, monitoring of vital signs including non-invasive estimation of blood pressure and heart rate, and the study of pain. There is currently a large body of literature contributing new knowledge on the relation of PPG pulse morphology, pulse wave analysis and pulse feature extraction with the physiological status of peripheral blood vessels, such as vascular aging and stiffness, blood pressure and compliance, microvascular disease, autonomic function, and others. There are also significant efforts in the utilization of the PPG for the detection of heart arrhythmias such as Atrial Fibrillation (AF). In addition, the recent significant contributions of PPG to wearable devices have had a major impact on the popularity and usability of PPG. Researchers continue to strive to combine the PPG sensory capabilities of wearables, such as smartwatches, with Artificial Intelligence (AI) machine learning approaches to deliver ubiquitous health monitoring solutions that go beyond currently available consumer devices. PPG and AI have a bright future together for the benefit of patients.

The aim of this Research Topic for Frontiers in Physiology is to bring together the latest cutting-edge basic and applied research in the field of Photoplethysmography. Our Research Topic comes from world-leading authors in the field and showcases 16 original research papers covering a diverse range of contributions in PPG measurement and analysis.

## Summary of published papers in this Research Topic

Cardiovascular disease continues to be the leading cause of death globally - this is one of the very important areas where PPG has considerable potential to help impact the burden of disease by allowing us to better understand vascular aging and enable low-cost, accessible monitoring of cardiovascular status. Djurić et al. in “*Using the photoplethysmography method to monitor age-related changes in the cardiovascular system*” collected single-site PPG measurements from above the left common carotid artery in 117 healthy adult participants (up to 70 years of age) and analyzed the data using a non-linear technique (detrended fluctuation analysis, DFA) to produce a ratio of scalar coefficients that were found to decrease exponentially with age–giving a biomarker for monitoring aging. Age-related changes in PPG shape have also been reported in the literature including the classification of the pulse into one of four classes based on the position of the dicrotic notch (Dawber et al., 1973). Zanelli et al. in “*Clustered photoplethysmogram pulse wave shapes and their associations with clinical data*” noted however that when working with real data, labeling waveforms into one of these four classes is no longer straightforward, but correct identification of the PPG shape could improve the precision and reliability of extracted biomarkers. Using a PPG dataset from 300 subjects (aged 19–83 years) the authors employed unsupervised machine learning and deep learning approaches to overcome the limitations of data labeling (including K-medoids-based clustering, a similarity matrix computed with Derivative Dynamic Time Warping, and PPG features extracted with CNN AutoEncoder). The results indicated that PPG waveforms do differ due to their dicrotic notch characteristics. However, there are additional differences such as the width of the systolic peak and the strength of a secondary systolic wave and by investigating the optimal number of clusters they found seven clusters of PPG wave shapes instead of the aforementioned four classes.

PPG provides a valuable way to study the dynamics of the cardiovascular system and key physiological variables such as blood pressure (BP) and heart rate (HR). Xing et al. in “*Temporal complexity in photoplethysmography and its influence on blood pressure*” used the Higuchi fractal dimension (HFD) and the autocorrelation function (ACF) to assess the temporal complexity of the PPG and interpreted the stochastic patterns with a model-based simulation which has the potential to help optimize BP estimation algorithms. The authors adapted the classic four-element Windkessel model to incorporate BP-dependent compliance profiles and simulations generated PPG responses at various time scales. Importantly, the relationship between complexity and hemodynamics predicted by their model aligned well with the experimental analysis of data collected from 40 healthy subjects. HFD and ACF had significant contributions to BP and displayed stability even in the presence of high cardiac output fluctuations. Temporal complexity patterns are essential for single-site PPG-based BP estimation and understanding the physiological implications of these patterns may aid in the development of such algorithms. A study of cardiovascular variability was also reported by Mejía-Mejía and Kyriacou with “*Spectral analysis for pulse rate variability assessment from simulated photoplethysmographic signals*”. Pulse rate variability (PRV) has been used as a surrogate for heart rate variability (HRV, measured via ECG) although it has been shown that there are differences that may result from physiological processes or from technical aspects of extracting PRV from PPG. The researchers extracted frequency-domain information from PRV in order to establish the best-performing combination of parameters and algorithms to obtain the spectral representation of PRV. They found that with specific interpolation methods, the Fast Fourier Transform (FFT) and multiple signal classification (PMUSIC) algorithms gave the best results, and considering the lower complexity of FFT over PMUSIC, it was recommended that FFT be considered as the appropriate technique to extract frequency-domain information from PRV signals.

**FIGURE 1:**
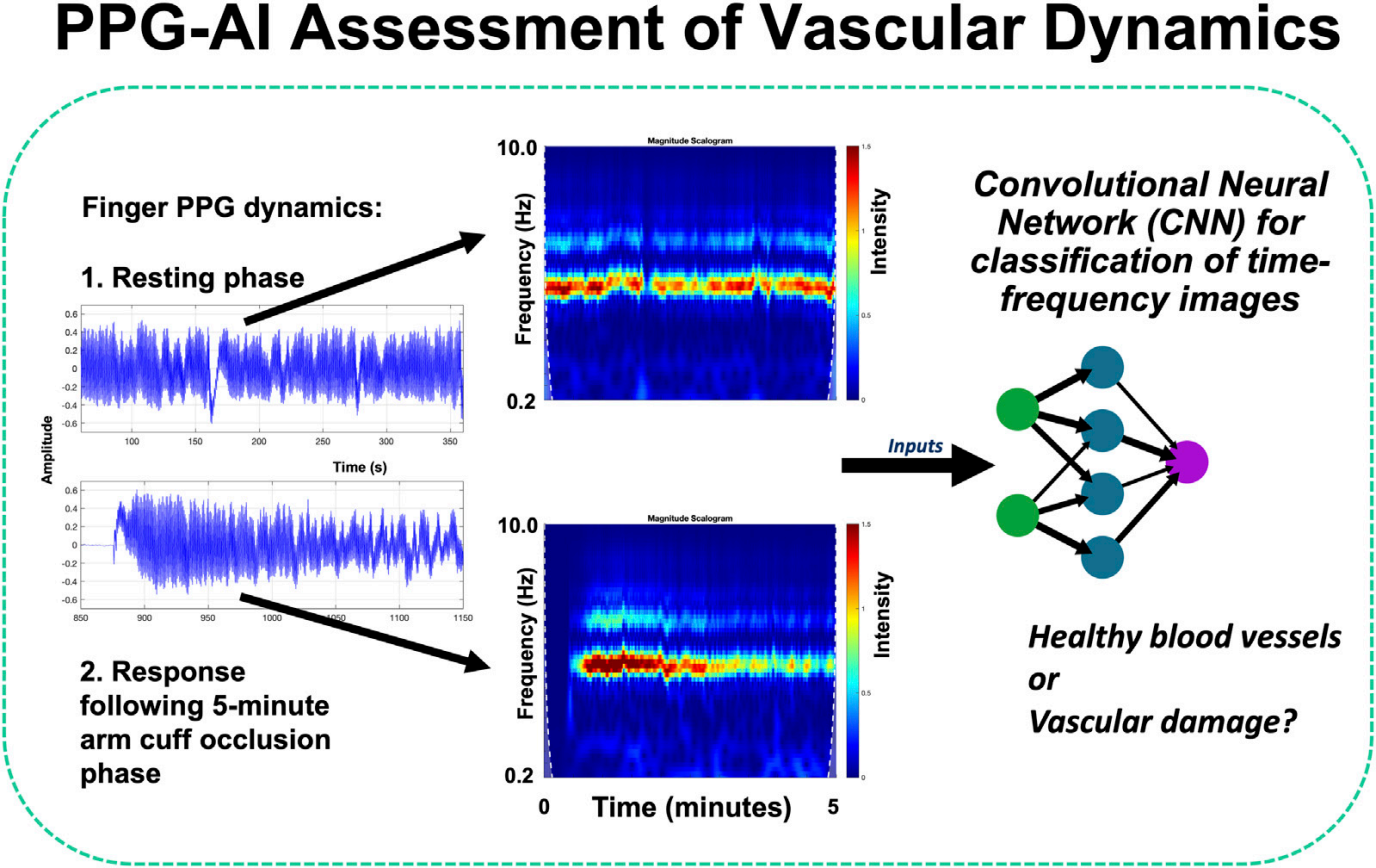
Advanced PPG sensing and analysis can give exciting new ways in to assess cardiovascular patients - including the study of vascular dynamics and signal variability (Kyriacou and Allen, 2021). For example, with AI, time-frequency image representations from combined resting and reactive hyperaemia PPG signals can be assessed using a pre-trained convolutional neural network (CNN) image classification system (Iqbal et al.).

The use of PPG for clinical monitoring was also covered in several leading-edge contributions. Stockwell et al. in “*Forehead monitoring of heart rate in neonatal intensive care*” described pioneering R&D in PPG sensor development for heart rate monitoring in critically unwell infants, with reflection mode measurements advantageously made at the forehead site rather than peripherally on a limb. They reported data comparing heart rates measured with a forehead-based PPG sensor against a wrist-based PPG sensor in 19 critically unwell infants in neonatal intensive care collecting 198 h of data, with good agreement between techniques (Bland-Altman limits of agreement of 8.44 bpm, bias −0.22 bpm) showing that the forehead is a reliable alternative location for measuring vital signs using the PPG. Roldan et al. in “*Non-invasive monitoring of intracranial pressure changes: healthy volunteers study*” aimed to evaluate the possible association between pulsatile near-infrared spectroscopic waveform features at the forehead and induced changes in intracranial pressure (ICP) in healthy volunteers. The authors reported data from 16 healthy volunteers with measurements acquired during changes in body position and during the Valsalva maneuver. The classification model features were extracted and an analysis was carried out to compare the two signals. The results revealed significant differences in the features extracted from these signals, demonstrating a correlation with ICP changes induced by position changes and the Valsalva maneuver. The classification models were able to identify changes in ICP using features from optical signals from the brain, with sensitivities ranging from 63% to 80% and specificities ranging from 60% to 70%; this work represents a first step toward non-invasive monitoring of intracranial pressure. Pettit et al. in “*Photoplethysmogram beat detection using Symmetric Projection Attractor Reconstruction*” presented a novel method that uses the Symmetric Projection Attractor Reconstruction (SPAR) method to generate an attractor in two-dimensional phase space from the PPG signal. A line was defined through the origin of this phase space as a Poincaré section, and beats were detected when the attractor trajectory crossed an optimally defined section. The method was assessed on the Wearable Stress and Affect Detection (WESAD) dataset and achieved median F1 scores of 74.3% in the Baseline phase, 63.0% during Stress, 93.6% during Amusement, and 97.7% during Meditation phases, comparable to one of the best algorithms identified in a recent benchmarking study of 15 beat detection algorithms. Iqbal et al. in “*Deep learning classification of systemic sclerosis from multi-site photoplethysmography signals*” described a pilot study assessing a novel approach to identify patients with the autoimmune connective tissue disease systemic sclerosis (SSc) using deep learning analysis of RGB scalograms of multi-site PPG waveforms (Figure 1 shows examples of multi-site PPG amplitude variability with illustrative analysis approach). Two different convolutional neural networks (CNNs, namely, GoogLeNet and EfficientNetB0) were trained and evaluated, with EfficientNetB0 showing overall better performance (87.3% accuracy) compared to GoogLeNet (83.1%) - both CNNs were superior to traditional ML methods.

A number of works in this Research Topic focused on non-invasive blood pressure (BP) measurement: “*Intensive care photoplethysmogram datasets and machine-learning for blood pressure estimation: Generalization not guaranteed*” (Weber-Boisvert et al.) studied the differences between the MIMIC waveform dataset and the PPG-BP dataset (an alternative public dataset obtained under controlled experimental conditions) and suggested that BP estimation models based on the MIMIC dataset have reduced predictive power in the general population; “*The identification of blood pressure variation with hypovolemia based on the volume compensation method*” (Chen et al.) studied the blood pressure variation, which is important in continuous blood pressure monitoring, especially in the case of low blood volume, and which is critical for survival; “*Towards continuous non-invasive blood pressure measurements—interpretation of the vasculature response to cuff inflation*” (Loureiro et al.) investigated BP surrogates (e.g., pulse transit or arrival time) and the results provide promising directions to improve the calibration process with cuff inflation toward accurate and robust non-invasive blood pressure estimation; “*Filtering-induced changes of pulse transmit time across different ages: a neglected concern in photoplethysmography-based cuffless blood pressure measurement*” (Liao et al.) showed that filtering-induced PTT changes are significantly influenced by age and PTT definition. These factors deserve further consideration to improve the accuracy of PPG-based cuffless blood pressure measurement using wearable sensors.

Several studies in this Research Topic addressed pain and its objective assessment: “*Induced pain affects auricular and body biosignals: From cold stressor to deep breathing*” (Rapalis et al.) examined targeted biofeedback parameters to close the loop in active pain therapy via auricular vagus nerve stimulation - personalizing pain therapy and increasing patient compliance; “*Photoplethysmography upon cold stress—impact of measurement site and acquisition mode*” (Fleischhauer et al.) systematically investigated the impact of the cold pressor test (CPT), i.e., a painful stimulus, on the morphology of PPG signals in 39 healthy volunteers and compared contact PPG recorded at the finger/earlobe with non-contact PPG (imaging PPG, iPPG) recorded at the face. The authors’ findings underlined the importance of the recording setup and physiological in addition to metrological differences related to the measurement protocol; “*Morphological features of the photoplethysmographic signal: a new approach to characterize the microcirculatory response to photobiomodulation*” (Ovadia-Blechman et al.) indicated that post-acquisition analysis of morphological features of the PPG waveform can provide new measures for investigating the microcirculatory response to photobiomodulation such as in the study of peripheral vasodilation, wound healing and pain; “*Contactless photoplethysmography for assessment of small fiber neuropathy*” (Marcinkevics et al.) also considered pain caused by small fiber neuropathy, seeking to develop objective non-invasive assessment methods. The team developed a modular prototype of a contactless (imaging) photoplethysmography system with three spectral bands (420, 540, and 800 nm) to assess peripheral neuropathy patients via a topical skin heating test and spectral analysis of cutaneous flow motion in 30 subjects, with results showing that neuropathic patients had a significantly lower vasomotor response (50%), flare area (63%), flare intensity index (19%), and neurogenic component (54%) of cutaneous flow motion compared to the control group, independent of photoplethysmography spectral band. iPPG has potential as a cost-effective alternative for the objective and non-invasive assessment of neuropathic patients, but further research is needed to enhance PPG signal quality and establish diagnostic criteria.

## Concluding remarks

We, the Editors, hope that this Research Topic will provide you with a deeper appreciation and understanding of PPG technology and its wide range of applications in clinical physiological measurement. We also hope that this Research Topic will help spark fresh ideas and new research collaborations across disciplines, including with biomedical engineering, and scientific and clinical colleagues. With the current trends in PPG-based technologies, sensing and analysis techniques, and clinical applications we can predict with great confidence that PPG will continue to grow and enable the development of further disruptive technologies for use in healthcare and well-being applications.
